# Multimodal MRI study of the relationship between plaque characteristics and hypoperfusion in patients with transient ischemic attack

**DOI:** 10.3389/fneur.2023.1242923

**Published:** 2023-09-28

**Authors:** Ying Sui, Jiali Sun, Yue Chen, Wei Wang

**Affiliations:** Department of MRI, The First Affiliated Hospital of Harbin Medical University, Harbin, China

**Keywords:** TIA, multimodal MRI, intracranial atherosclerotic, ABCD^2^ score, high-resolution MRI

## Abstract

**Objective:**

Transient ischemic attack is a significant risk factor for acute cerebral infarction. Previous studies have demonstrated that hypoperfusion in patients with transient ischemic attack was associated with the recurrence of transient ischemic attack, stroke, and persistent worsening of neurological symptoms. Moreover, transient ischemic attack patients classified as high-risk group according to the ABCD^2^ score have a higher incidence of stroke. Therefore, the objective of this study was to investigate the plaque characteristics of transient ischemic attack patients with concomitant cerebral hypoperfusion using multimodal MRI, as well as hemodynamic changes in the high-risk group with transient ischemic attack patients.

**Materials and methods:**

A total of 151 patients with transient ischemic attack were prospectively recruited for this study. All enrolled patients underwent multimodal MRI, including DWI, TOF-MRA, HR-VWI, and DSC-PWI. Finally, 56 patients met the inclusion criteria. Based on DSC-PWI images, patients were divided into two groups: hypoperfusion (*n* = 41) and non-hypoperfusion (*n* = 15). Clinical baseline characteristics and plaque characteristics were analyzed between the two groups. Furthermore, within the hypoperfusion group, patients were further classified into low-risk (*n* = 11) and high-risk (*n* = 30) subgroups based on the ABCD^2^ score. Hemodynamic differences between these subgroups were also analyzed.

**Results:**

Compared with the non-hypoperfusion group, the hypoperfusion group had a significantly higher prevalence of hypertension (68.3% vs. 33.3%, *p* = 0.019) and hyperhomocysteinemia (65.9% vs. 33.3%, *p* = 0.029). Moreover, the hypoperfusion group exhibited more significant luminal stenosis degree [41.79 ± 31.36 vs. 17.62 ± 13.62, *p* = 0.006] and greater NWI (57.1% ± 20.47% vs. 40.21% ± 21.56%, *p* = 0.009) compared to the non-hypoperfusion group. In addition, the high-risk group identified by the ABCD^2^ score had a higher rMTT [117.6(109.31–128.14) vs. 108.36(100.67–119.92), *p* = 0.037].

**Conclusion:**

Transient ischemic attack patients with hypoperfusion exhibited a higher prevalence of hypertension and hyperhomocysteinemia, as well as higher luminal stenosis degree, and greater NWI. Furthermore, Transient ischemic attack patients in the high-risk group demonstrated higher MTT.

## Introduction

Transient ischemic attack (TIA) is an episode of transient neurological dysfunction without evidence of acute cerebral infarction (1). In Asian populations, intracranial atherosclerotic disease, particularly middle cerebral artery (MCA) atherosclerosis, is the most common cause of acute ischemic stroke and TIA (2, 3). Approximately half of TIA patients experience a stroke within the first 48 h, and the rate of stroke within 90 days can reach 10%–15% (4). Therefore, rapid and effective risk stratification is crucial in identifying TIA patients who may be at risk of ischemic stroke at an early stage. This will enable clinicians to prioritize high-risk patients who require urgent management. In clinical, TIA is a significant risk factor for acute cerebral infarction. Studies have shown that the progressive development of intracranial atherosclerotic plaques leads to the narrowing of the blood vessel lumen, ultimately resulting in a decrease in cerebral blood flow (5). Meanwhile, decreased cerebral perfusion in TIA patients, even in the absence of an acute infarct lesion, may be associated with the recurrence of TIA, stroke, and persistent worsening of neurological symptoms (6). Additionally, the ABCD^2^ score (named according to five factors: age, blood pressure, clinical characteristics, symptom duration, and diabetes) is a widely used risk prediction tool to determine the risk of early progression to ischemic stroke in TIA patients (1, 7). The ABCD^2^ score has been found to be positively correlated with the incidence of cerebral infarction within 1 week after TIA, meaning that the higher the score, the higher the incidence of cerebral infarction in the short term (8). Therefore, TIA patients are divided into a low-risk (≤3 points) group and a high-risk (≥4 points) group according to the ABCD^2^ score (1). Elucidating the mechanisms and predictors of hypoperfusion in TIA patients and understanding the hemodynamics in patients at high risk for TIA is critical.

High-resolution vessel wall imaging (HR-VWI) is widely used to assess the morphology of intracranial atherosclerotic plaques, allowing noninvasive visualization of the wall and lumen of the MCA. It helps explore the distribution characteristics and anatomy of the plaque, aiding in understanding the pathophysiological features of atherosclerosis (9). HR-VWI can also help identify high-risk patients who require intensive treatment by measuring atherosclerotic plaque burden and instability, as well as monitor treatment effects (10). Furthermore, dynamic susceptibility contrast perfusion-weighted imaging (DSC-PWI) is a unique dynamic imaging technique that changes the magnetization rate of tissue and, thus, the strength of the MRI signal over time by intravenous contrast injection to observe the intracranial hemodynamic state (11). Multimodal MRI imaging provides an essential foundation for understanding the pathogenesis and risk stratification of patients with TIA, enabling the development of individualized treatment plans for precision medicine.

The differences in plaque features between ischemic stroke and TIA patients, as well as symptomatic and asymptomatic patients, have been extensively studied (12–14). However, there have been few studies comparing the distinctions in plaque characteristics between hypoperfusion and non-hypoperfusion in TIA patients. It remains unclear whether plaque characteristics differ in TIA patients with and without hypoperfusion. Therefore, the objective of this study was to investigate the plaque characteristics of TIA patients with concomitant cerebral hypoperfusion using multimodal MRI, as well as hemodynamic changes in the high-risk group with TIA patients.

## Materials and methods

### Study participants and clinical data

The study protocol was approved by the Ethics Committee of The First Hospital of Harbin Medical University . All patients provided either written or verbal consent to participate in the study. The study was conducted in accordance with the Declaration of Helsinki (as revised in 2013). Prospective inclusion of patients with atherosclerotic TIA in the MCA, treated at The First Hospital of Harbin Medical University, took place from October 2021 to July 2023 . The inclusion criteria were as follows: (1) age ≥ 18 years; (2) DWI images were negative; (3) patients had multiple (≥2) TIA symptoms in the past, with each attack lasting no more than 24 h; (4) contralateral limb numbness or weakness at the MCA stenosis in TIA patients; (5) MR examination performed within 7 days after onset; (6) presence of at least one atherosclerotic risk factor, such as hypertension, diabetes mellitus, dyslipidemia, alcohol consumption, or smoking. The exclusion criteria were as follows: (1) acute cerebral infarction confirmed by DWI; (2) contraindications to MRI enhancement; (3) previous history of cerebral infarction, cerebral hemorrhage, brain surgery, and cerebrovascular intervention; (4) combined ipsilateral carotid artery (ICA) stenosis ≥ 50%; (5) not due to atherosclerotic diseases, such as dissection, vasculitis, moyamoya disease, or cardiogenic embolism; (6) poor MRI image quality that prevented further analysis.

Clinical baseline characteristics and multimodal MR imaging of the patients were collected for the patients. According to the DSC-PWI images, the patients were divided into two groups: hypoperfusion and non-hypoperfusion. Furthermore, within the hypoperfusion group, segmented into low-(0–3) and high-(4–7) risk groups were performed based on the ABCD^2^ score (1).

Demographic and clinical characteristics were collected from clinical records, including age, gender, BMI, history of hypertension, diabetes, total cholesterol, triglycerides, LDL, HDL, and hyperhomocysteinemia. Hypertension was defined as self-reported history of hypertension, mean blood pressure ≥ 140/90 mmHg, or taking anti-hypertensive medication (15); diabetes was defined as self-reported use of insulin or oral hypoglycemic drugs, or HbA1c ≥ 6.5% (16); total cholesterol ≥ 5.2 mmol/L, triglycerides ≥ 1.7 mmol/L, LDL ≥ 3.7 mmol/L, HDL ≤ 1.04 mmol/L, or self-reported history of dyslipidemia (17), and hyperhomocysteinemia defined as >15 μ mol/L (18). History of smoking: ≥1 cigarette per day for six consecutive months; history of alcohol consumption: >2 U for men and >1 U for women on average per day for the past year (19).

### MRI data acquisition

A 3.0 T MRI scanner (Achieva; Philips Healthcare, The Netherlands) with a 32-channel phased-array coil was used. Patients were trained to breathe before scanning to reduce the effect of motion artifacts on image quality. The scanning protocol and parameters included diffusion-weighted imaging (DWI), TR/TE 21/3.45 ms, thickness 0.6 mm, Flip angle 20°, FOV 194 mm × 194 mm; three-dimensional time-of-flight magnetic resonance angiography (3D-TOF-MRA), TR/TE 1,927/55 ms, thickness 4 mm, Flip angle 90°, FOV 230 mm × 230 mm; HR-VWI, and DSC-PWI. HR -VWI including T1-weighted three-dimensional volumetric isotropic turbo spin echo acquisition (T1-3D-VISTA), TR/TE 800/18 ms, thickness 0.6 mm, Flip angle 90°, FOV 180 mm × 144 mm; simultaneous non-contrast angiography and intraplaque hemorrhage (SNAP), TR/TE 10/5.8 ms, thickness 0.6 mm, Flip angle 11°, FOV 160 mm × 160 mm; T2-weighted three-dimension volumetric isotropic turbo spin echo acquisition (T2-3D-VISTA), TR/TE 2,400/90 ms, thickness 0.6 mm, Flip angle 90°, FOV 250 mm × 250 mm; and enhanced T1-3D-VISTA. The contrast agent was Gd-DTPA contrast, using 0.1 mL/kg. DSC-PWI, TR/TE 1,795/40 ms, thickness 0.6 mm, Flip angle 75°, FOV 224 mm × 224 mm, was performed by injecting Gd (0.1 mL/kg) via the anterior elbow vein at a rate of 5 mL/s with a high-pressure syringe, followed by flushing 20 mL of saline at the same rate and acquiring planar gradient echoes every 2 s (T2^*^) images for the 60s.

### MRI image analysis

Two radiologists with more than 2 years of diagnostic experience conducted qualitative and quantitative analyses of the images while remaining blind to the clinical data of all recruited patients. In case of any disagreements between the observers, a consensus was reached through a joint reading. All parameter measurements were performed on the Philips Intellispace Portal workstation. The quality of MR images was classified as poor, marginal, good, and excellent according to the signal-to-noise, and images graded as poor were excluded from the study. On HR-VWI images, atherosclerotic plaques exhibited eccentric thickening of the vessel wall (17). According to the symptoms of TIA, the location of diseased blood vessels (left/right MCA) was defined. Plaque was defined as eccentric thickening of MCA wall. A culprit plaque was defined as the layer of the maximum lumen narrowing (MLN) of the MCA (20, 21). The reference lumen was the plaque-free segment proximal or distal to the largest luminal stenosis. The short axial T1-3D-VISTA images were magnified to 200% and the outer wall area (OWA) and inter wall area (IWA) were manually measured at the MLN site and the reference site. Intraplaque hemorrhage (IPH) was defined when the signal intensity (SI) of the culprit plaque was higher than 150% of gray matter (22). All cross-sections with plaque were categorized in accordance with whether the plaque was centered on the superior, inferior, dorsal, or ventral side of the vessel. If a plaque was distributed between two quadrants, the quadrant with the thickest plaque was selected (23). The plaque measurement is as follows: wall area (WA) = OWA-IWA; plaque area (PA) was calculated as WA*_MLN_*-WA*_reference_* (24); the degree of stenosis = (1 − IWA*_MLN_*/IWA*_reference_*) × 100% (12). The measurement of plaque burden is expressed by the normalized wall index (NWI), which is defined as WA*_MLN_*/OWA*_MLN_* × 100% (25); remodeling index (RI) = OWA*_MLN_*/OWA*_reference_* (RI > 1.05 was defined as positive remodeling and RI < 0.95 was defined as negative remodeling) (21). Additionally, SI was quantified with manually drawn regions of interest on HR-VWI images. The signal intensity of gray matter (SI*_graymatter_*) was measured by manually drawing a round region of 8–10 mm^2^ at the adjacent normal gray matter on matched pre and post-contrast T1-weighted images, respectively. The contrast enhancement ratio = [(SI*_post-plaque_*/SI*_post-graymatter_*) − (SI*_pre-plaque_*/SI*_pre-graymatter_*)]/(SI*_pre-plaque_*/SI*_pre-graymatter_*) × 100% (26).

The DSC-PWI sequence was utilized to generate pseudo-color maps of region cerebral blood flow (rCBF), region cerebral blood volume (rCBV), mean transit time (MTT), and time to peak (TTP). It is challenging to compare hemodynamic parameters due to individual differences. Therefore, to avoid bias in the data, the relative rCBF (rrCBF), relative rCBV (rrCBV), relative MTT (rMTT), and relative TTP (rTTP) were calculated based on the affected/mirror contralateral side. Since there is no universally validated or consistent perfusion value for distinguishing risky from benign tissue. We based on previous research that, in the pseudo-color map, the hypoperfusion group was defined as reduced perfusion on the affected side compared with the contralateral side, with rrCBF < 75%. The non-hypoperfusion group was defined as no abnormal perfusion areas observed on the pseudo-color chart by visual observation (27, 28).

### Statistical analysis

The statistical analysis of the data was performed using IBM SPSS 26.0 software. The Kolmogorov–Smirnov test was used to analyze the normality of continuous variables. Variables that followed a normal distribution were expressed as mean ± standard deviation (SD), and comparisons between groups were conducted using independent Student’s *t*-test. For variables with skewed distributions, median and interquartile range (IQR) were used, and Mann–Whitney *U*-test for comparison between groups. Categorical variables were represented as numbers and frequencies (%), and group comparisons were carried out using the chi-square test. In the chi-square test, if the expected count of any cell was less than 5, Pearson’s Chi-square test was applied. If any cell had an expected count was less than 5, a continuity correction test was used. If the minimum expected count was less than 1, Fisher’s exact test was employed . The interobserver reproducibility of continuous variables was assessed using intra-group correlation coefficients (ICC) and the agreement was classified as very good (r > 0.75), good (r = 0.4–0.75), or poor (r < 0.4). Cohen’s kappa was utilized to determine the interobserver agreement in identifying plaque distribution. The *p* < 0.05 difference was statistically significant.

## Results

### Demographic and clinical information

**Figure 1:**
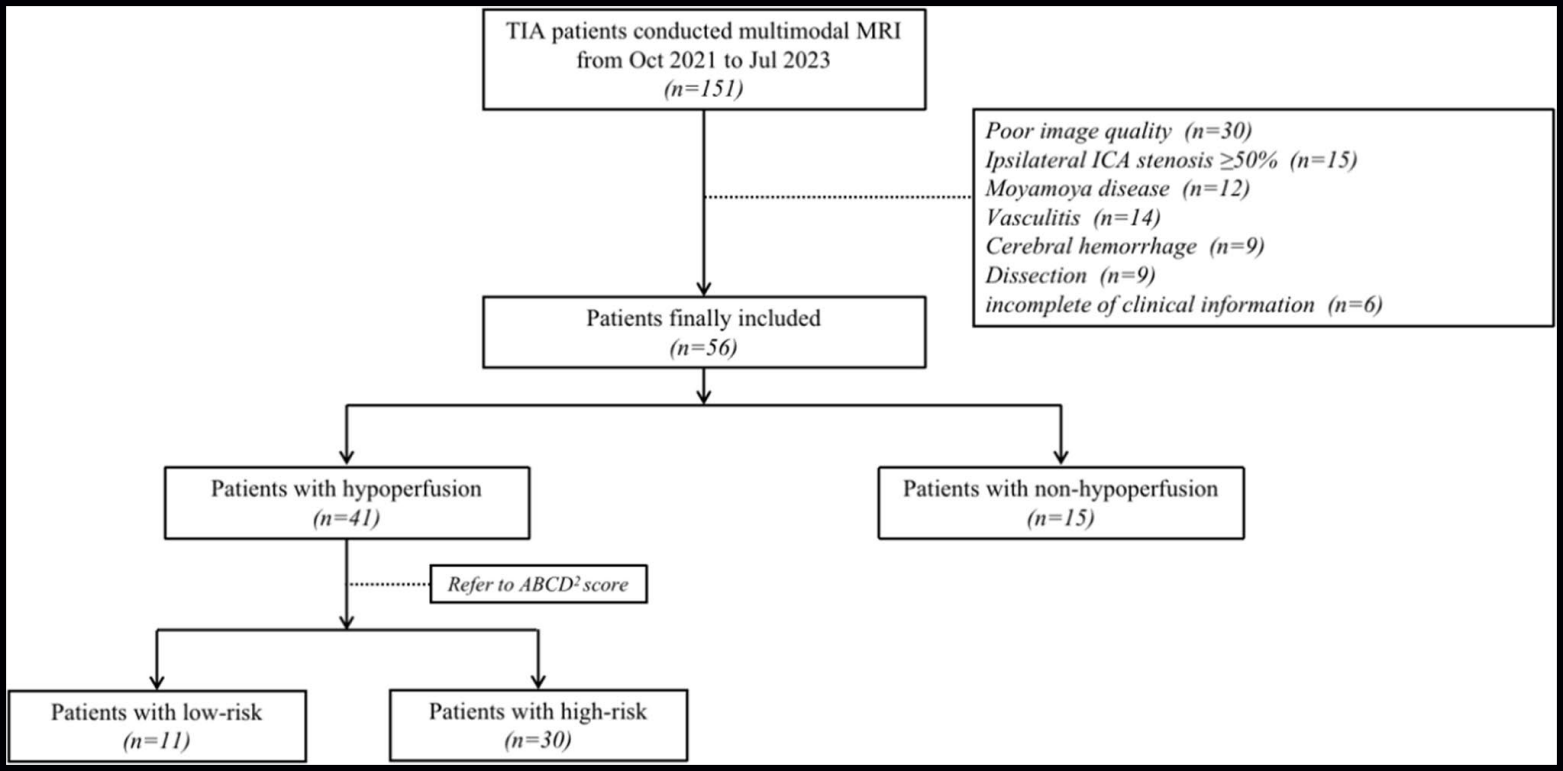
Flow chart showing process of study population selection. TIA, transient ischemic attack; ICA, internal carotid artery.

The patient selection process is summarized in Figure 1. During the trial period, 151 patients with TIA were prospectively recruited for MRI. Among the recruited patients, 95 patients were excluded due to the following reasons: poor image quality (*n* = 30); ipsilateral ICA stenosis ≥ 50% (*n* = 15), moyamoya disease (*n* = 12), vasculitis (*n* = 14), cerebral hemorrhage (*n* = 9), dissection (*n* = 9), and incomplete of clinical information (*n* = 6). Ultimately, 56 patients with a mean age of 53.45 ± 11.86 years were included in this study. The hypoperfusion group consisted of 41 patients (mean age 55 ±11 years, 21 male), while the non-hypoperfusion group consisted of 15 patients (mean age 50 ± 14 years, 10 male).

Among the 56 patients with TIA, the prevalence of hypertension was 58.92%, diabetes mellitus was 32.14%, hyperhomocysteinemia was 57.14%, and dyslipidemia was 37.5%. In addition, 20 patients (35.71%) had a history of smoking, and 13 patients (23.21%) had a history of alcohol consumption. In the hypoperfusion group, the proportion of patients with hypertension (68.3% vs. 33.3%) and hyperhomocysteinemia (65.9% vs. 33.3%) was significantly higher than that in the non-hypoperfusion group, and the difference was statistically significant (*p* = 0.019, *p* = 0.029). There were no significant differences between the two groups in terms of gender, age, BMI, diabetes, cholesterol, triglycerides, LDL, HDL, smoking, and alcohol consumption (*p* > 0.05). The clinical characteristics of TIA patients in the hypoperfusion and non-hypoperfusion groups were compared in Table 1.

**Table 1:** The comparison of clinical baseline characteristics between the two groups.

	Hypoperfusion (*n* = 41)	Non-hypoperfusion(*n* = 15)	*t/Z/* χ *^2^*	*p*
Male, *n* (%)	21(51.2)	10(66.7)	1.06*^c^*	0.303
Age (years)	55 ± 11	50 ± 14	1.377*^a^*	0.174
BMI (kg/m^2^)	25.39(22.86–28.15)	26.12(21.3–28.41)	−0.352*^b^*	0.725
Hypertension, *n* (%)	28(68.3)	5(33.3)	5.546*^c^*	0.019^*^
Diabetes mellitus, *n* (%)	15(36.6)	3(20)	0.729*^c^*	0.393
Cholesterol (mmol/L)	3.71(3.25–4.55)	3.77(3.17–5.34)	−0.537*^b^*	0.592
Triglycerides (mmol/L)	1.43(1.1–1.88)	1.75(1.24–1.98)	−0.842*^b^*	0.4
LDL (mmol/L)	1.95 ± 0.22	1.87 ± 0.35	0.871*^a^*	0.395
HDL (mmol/L)	1.49 ± 0.51	1.53 ± 0.52	−0.297*^a^*	0.768
Hyperhomocysteinemia, *n* (%)	27(65.9)	5(33.3)	4.743*^c^*	0.029^*^
Smoking, *n* (%)	16(39)	4(26.7)	0.73*^c^*	0.393
Alcohol consumption, *n* (%)	9(22)	4(26.7)	0*^c^*	0.99

Data are presented as mean ± SD, median [interquartile range (IQR)], or *n* (%). *p* < 0.05 indicates significant difference. BMI, body mass index; LDL, low-density lipoprotein; HDL, high-density lipoprotein. *^a^*, *p*-value was obtained by *t*-test; *^b^*, *p*-value was obtained by Mann–Whitney *U*-test; *^c^*, *p*-value was obtained by chi-square test; ^*^, the difference was statistically significant.

### Interreader reproducibilities

Good interreader reproducibilities were found: IWA*_MLN_* (ICC 0.997, 95% CI 0.994–0.998); IWA*_reference_* (ICC 0.91, 95% CI 0.985–0.995); OWA*_MLN_* (ICC 0.994, 95% CI 0.990–0.997); OWA*_reference_* (ICC 0.988, 95% CI 0.980–0.993); SI*_pre-plaque_* (ICC 0.954, 95% CI 0.923–0.973); SI*_pre-graymatter_* (ICC 0.878, 95% CI 0.801–0.927); SI*_post-plaque_* (ICC 0.946, 95% CI 0.910–0.968); SI*_post-graymatter_* (ICC 0.880, 95% CI 0.803–0.928); rrCBF (ICC 0.760, 95% CI 0.623–0.852); rrCBV (ICC 0.795, 95% CI 0.674–0.874); rMTT (ICC 0.802, 95% CI 0.684–0.879); rTTP (ICC 0.758, 95% CI 0.618–0.851); plaque distribution *k* = 0.818; IPH *k* = 0.707.

### Plaque characteristics between hypoperfusion and non-hypoperfusion groups

**Figure 2:**
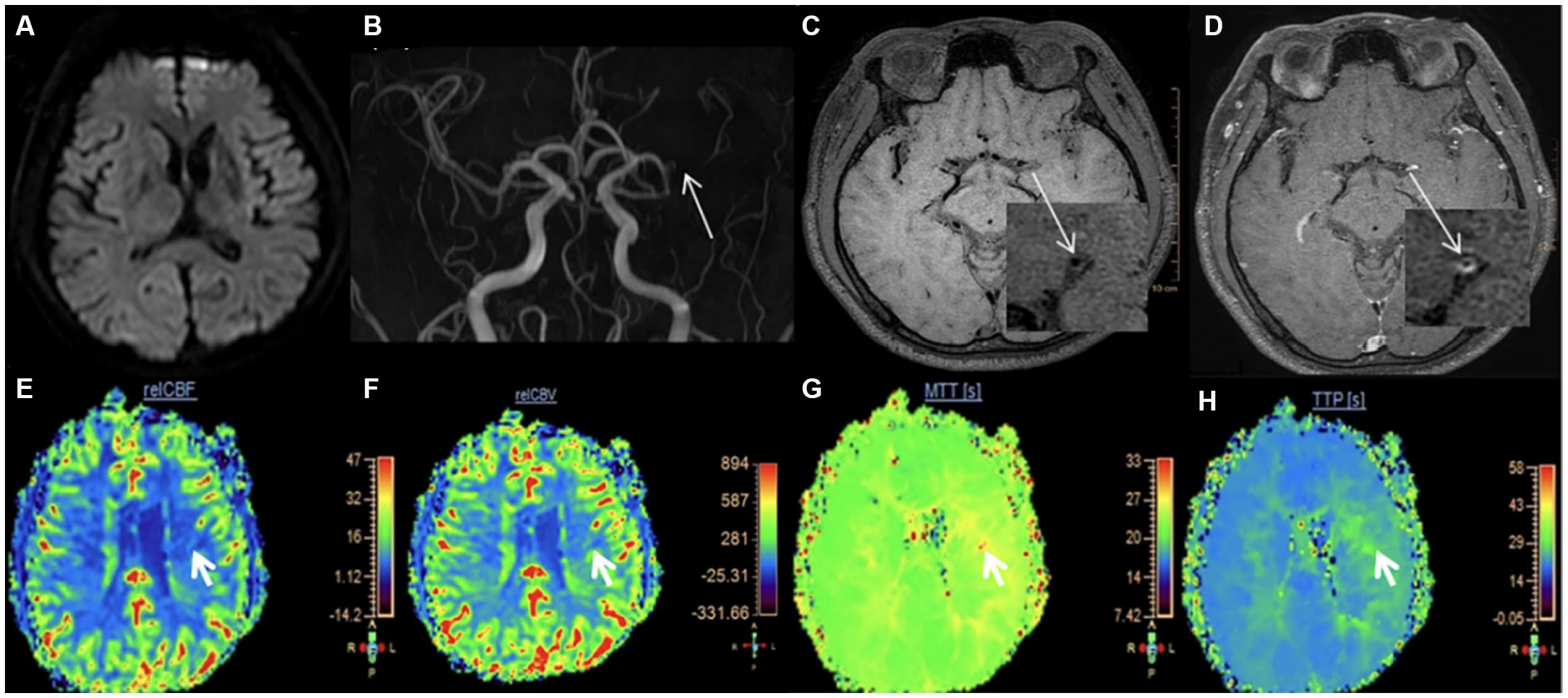
Measurement of vessel and plaque characteristics in hypoperfusion. A 32-year-old male was admitted to our hospital for 4 h due to right limb weakness. **(A)** DWI is normal; **(B)** The stenotic site of the left MCA. The white arrow shows the stenotic site of the left MCA; **(C)** T1-weighted HR-VWI imaging shows the plaque in the left MCA. The white arrow shows the short-axis view; **(D)** Enhanced T1-weighted HR-VWI imaging showing the measurement at the most narrowed site in left MCA. OWA = 2.04 mm^2^, IWA = 0.89 mm^2^. **(E–H)** The four pseudo-color maps of rCBF, rCBV, MTT, and TTP are shown respectively, with the white arrow indicating areas of hypoperfusion. rCBF = 5.86, rCBV = 156.84, MTT = 26.78 s, TTP = 26.84 s, rrCBF = 71.19%. DWI, diffusion-weighted imaging; MCA, middle cerebral artery; HR-VWI, high-resolution vessel wall imaging; OWA, outer wall area; IWA, inter wall area.

**Figure 3:**
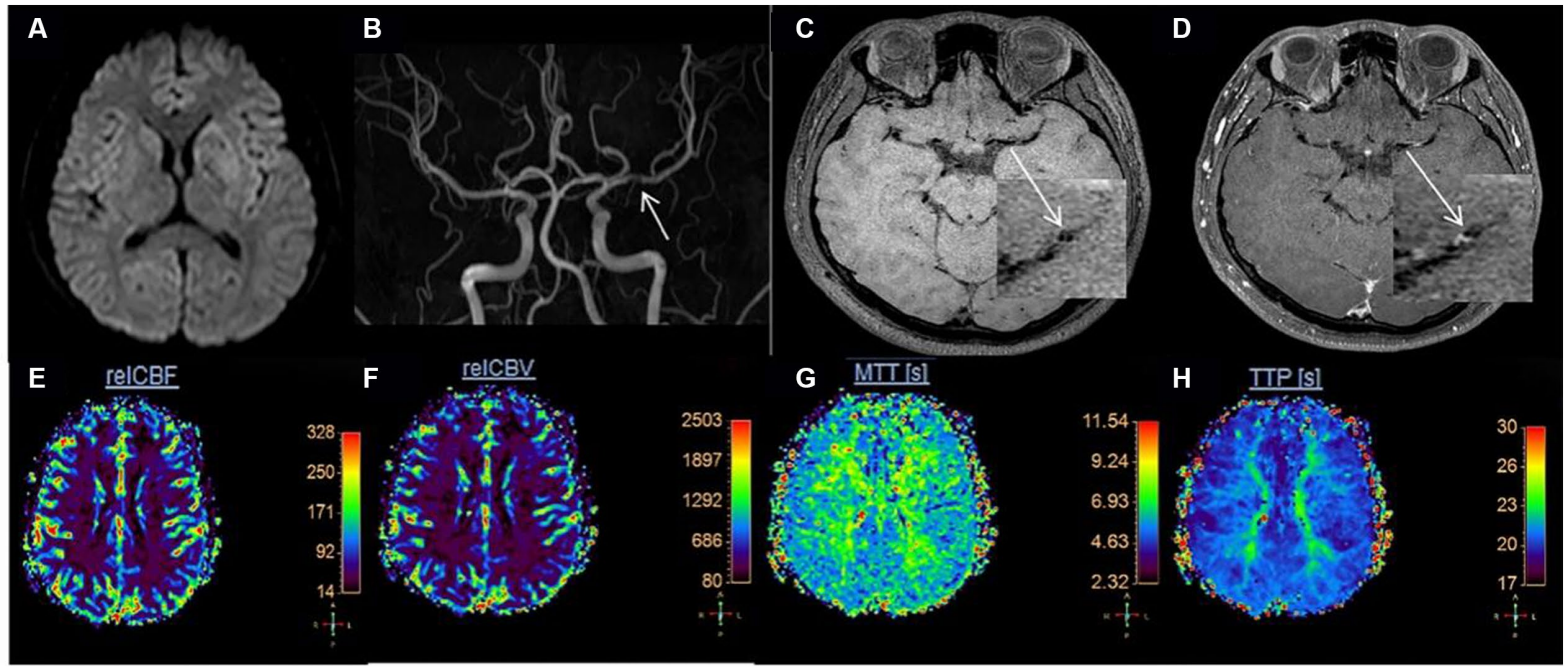
Measurement of vessel and plaque characteristics in non-hypoperfusion. Female, 59 years old, left upper limb weakness for more than 3 h. **(A)** DWI is normal; **(B)** The stenotic site of the left MCA. The white arrow shows the stenotic site of the left MCA; **(C)** T1-weighted HR-VWI imaging shows the plaque in the left MCA. The white arrow shows the short-axis view; **(D)** Enhanced T1-weighted HR-VWI imaging showing the measurement at the most narrowed site in left MCA. OWA = 5.19 mm^2^, IWA = 1.33 mm^2^. **(E–H)** Pseudo-color maps of rCBF, rCBV, MTT, and TTP were shown respectively, and none of them revealed the region of hypoperfusion. DWI, diffusion-weighted imaging; MCA, middle cerebral artery; HR-VWI, high-resolution vessel wall imaging; OWA, outer wall area; IWA, inter wall area.

**Figure 4:**
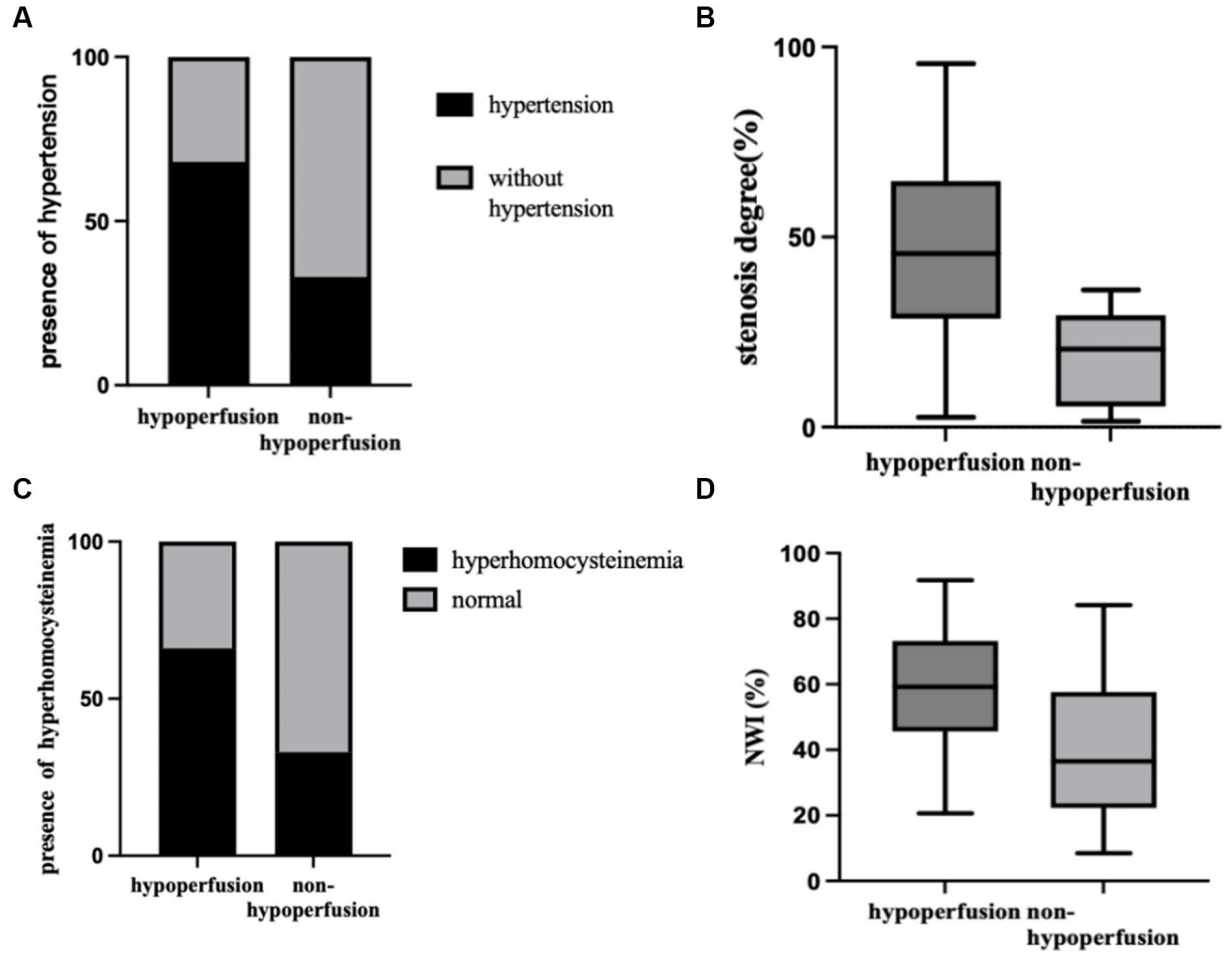
Comparison between hypoperfusion and non-hypoperfusion in prevalence of hypertension **(A)**, hyperhomocysteinemia **(B)**, stenosis degree **(C)**, and NWI **(D)**. All were statistically different. NWI, normalized wall index.

Measurement of vessel and plaque characteristics in hypoperfusion and non-hypoperfusion is shown in Figures 2, 3, respectively. In the hypoperfusion group, both luminal stenosis (41.79% vs. 17.62%) and NWI (57.1 % vs. 40.21) were significantly higher than in the non-hypoperfusion group, and the differences were statistically significant (*p* = 0.006,0.009). The remaining plaque characteristics did not significantly differ between the two groups (all *p* > 0.05). The comparison of plaque characteristics between the two groups is presented in Table 2. Figure 4 illustrates the differences in clinical baseline characteristics and plaque characteristics between the two groups.

**Table 2:** The comparison of plaque characteristics between the two groups.

	Hypoperfusion (*n* = 41)	Non-hypoperfusion (*n* = 15)	*t/Z/* χ *^2^*	*p*
IWA*_MLN_* (mm^2^)	1.06(0.76–1.82)	3.1(0.97–5.23)	−1.842*^b^*	0.065
IWA*_reference_* (mm^2^)	2.21(1.68–3.25)	3.63(1.33–5.4)	−0.157*^b^*	0.875
OWA*_MLN_* (mm^2^)	3.42(2.08–5.27)	4.78(2.04–7.42)	−0.712*^b^*	0.476
OWA*_reference_* (mm^2^)	4.52(3.4–6.88)	5.58(2.25–9.08)	−0.231*^b^*	0.817
PA (mm^2^)	0.98 ±0.93	0.91 ± 0.7	0.186*^a^*	0.853
Stenosis degree (%)	41.79 ± 31.36	17.62 ± 13.62	2.875*^a^*	0.006^*^
Contrast enhancement ratio (%)	150.77(92.1–257.7)	157.15(101.06–236.7)	−0.009*^b^*	0.993
RI	69.66(54.88–90.94)	83.92(71.62–91.6)	−1.915*^b^*	0.055
IPH, *n* (%)	9(22)	1(6.67)	0.862*^c^*	0.353
NWI (%)	57.1 ± 20.47	40.21 ± 21.56	2.697*^a^*	0.009^*^
**Distribution, n (%)**				
Superior	8(19.51)	2(13.33)	0.02*^c^*	0.888
Inferior	21(51.22)	5(33.33)	1.413*^c^*	0.235
Ventral	8(19.51)	4(26.67)	0.044*^c^*	0.834
Dorsal	4(9.76)	4(26.67)	1.37*^c^*	0.242

Data are presented as mean ± SD, median interquartile range (IQR), or *n* (%). PA, plaque area; IPH, intra-plaque hemorrhage; NWI, normalized wall index. *^a^*, *p*-value was obtained by *t*-test; *^b^*, *p*-value was obtained by Mann–Whitney *U*-test; *^c^*, *p*-value was obtained by Chi-square test; ^*^, the difference was statistically significant.

### Comparison of hemodynamic between the high-risk and low-risk groups

Patients in the hypoperfusion group were divided into low-risk and high-risk groups according to the ABCD^2^ score. The rMTT was higher in the high-risk group [108.36(100.67–119.92)] than the low-risk group [117.6(109.31–128.14)], and the difference was statistically significant (*p* = 0.037). The hemodynamic comparison between the low-risk and high-risk groups is shown in Table 3.

**Table 3:** The comparison of perfusion parameters between subgroups.

	Low-risk (*n* = 11)	High-risk (*n* = 30)	*t/Z*	*p*
rrCBF (%)	54.41 ± 13.79	46.77 ± 18.88	1.224*^a^*	0.228
rrCBV (%)	66.82(55.6–71.79)	59.79(38.49–75.98)	−0.147*^b^*	0.896
rMTT (%)	108.36(100.67–119.92)	117.6(109.31–128.14)	−2.089*^b^*	0.037
rTTP (%)	110.59(102.75–113.65)	114.78(104.78–122.68)	−1.196*^b^*	0.241

*^a^*, *p*-value was obtained by *t*-test; *^b^*, *p*-value was obtained by Mann–Whitney *U*-test.

## Discussion

TIA serves as a vital warning indicator of impending stroke (29). Intracranial atherosclerotic significantly contributes to both vascular events (30). Prior studies have highlighted the persistent high risk faced by TIA patients in the early stages (31). Multimodal MRI enables the observation of changes in brain structure and function from various perspectives, facilitating the early identification of cerebral ischemic states and etiology. This, in turn, aids in the accurate stratification of risk among patients with cerebrovascular disease and helps identify high-risk groups for personalized and precise treatment to mitigate risk. To the best of our knowledge, this is the first study to use HR-VWI combined with DSC-PWI to assess plaque characteristics in TIA patients with reduced cerebral blood flow and examine hemodynamic changes in high-risk TIA patients.

Our results found that patients with hypoperfusion had a significantly higher prevalence of hypertension and hyperhomocysteinemia compared to those without hypoperfusion. These findings align with previous research (32, 33), and Wang et al. concluded that hypertension is an independent risk factor for perfusion abnormalities in TIA patients (34). Hypertension has a substantial impact on cerebral circulatory changes, as repeated mechanical stress, degradation of elastin fibers in the vascular wall, and alteration of vascular endothelial function or morphology caused by hypertension contribute to reduced cerebral perfusion through increased cerebrovascular resistance and impaired self-regulation (35). Another possible explanation is that angiotensin II (Ang II), the primary hormone of the renin-angiotensin system (RAS), plays a vital role in the pathophysiology of hypertension. Ang II affects cerebral blood flow through a cascade of vasoconstriction and endothelial dysfunction (36). In addition, hyperhomocysteinemia is associated with the formation of atherosclerotic plaque, platelet aggregation, and adhesion. It can also disrupt vascular endothelial cells, vascular elastic fibers, and flow regulation, adversely influencing vascular regeneration and circulation. Wang et al. have also discovered that hyperhomocysteinemia expression promotes vascular stenosis (37). Furthermore, this study revealed that patients with more significant stenosis are more likely to develop hypoperfusion. Proximal arterial stenosis can transmit the slowdown effects on blood flow to the downstream areas of the brain parenchyma; reduced cerebral blood flow leads to decreased perfusion (38). As a result, the higher the degree of luminal stenosis, the lower the cerebral blood flow and the higher the risk of ischemic cerebrovascular events. Consequently, TIA with luminal stenosis should be given high prioritized. Studies have demonstrated that the relationship between NWI and luminal stenosis is primarily linear (39). Moreover, Jia et al. have shown that NWI is the most effective index for evaluating the severity of atherosclerosis and more accurately reflects plaque progression (40). Additionally, NWI progression is a significant independent predictor of recurring symptomatic ischemic events. Therefore, according to these data, we can infer that a larger NWI in TIA patients is more likely to lead to adverse cerebrovascular events.

The ABCD^2^ score performed well in identifying patients at high risk of TIA and was frequently used to predict TIA recurrence (41, 42). DSC-PWI is a functional imaging approach that can sensitively reflect the blood perfusion of brain tissue and can also semi-quantitatively study the changes in blood flow. However, in patients with negative DWI and focal hypoperfusion on DSC-PWI, there was a high risk of stroke recurrence in the abnormal perfusion area after 7 days (43). Abnormal hemodynamics inhibit cerebrovascular self-regulation, decreasing vascular reserve and increasing the risk of stroke (44). Therefore, the brain’s ability to regulate blood flow to meet metabolic demands and compensate for cerebral perfusion is an important protective mechanism against the development of cerebral ischemia. In this study, the patients in the hypoperfusion group were divided into low-risk and high-risk groups based on the ABCD^2^ score, which helped clarify the hemodynamics of the high-risk group for early detection and timely treatment. This study found that both the high-risk group and the low-risk group experienced perfusion changes, which may be attributed to the decompensation of the blood supply in the cerebrovascular trunk, leading to a reduction in cerebral perfusion pressure through self-regulation and resulting in abnormal in local hemodynamics and a decrease in distal vascular resistance. As a result, collateral circulation compensation becomes the primary mechanism. Furthermore, the high-risk group exhibited more pronounced MTT prolongation compared to the low-risk group. MTT, defined as the time required for the contrast agent to pass through the microcirculation, is highly sensitive to perfusion differences (45). This suggests that the high-risk group had poorer microcirculation status. Therefore, it is further suggested that patients in the high-risk group may require more active and comprehensive interventions to maintain the normal metabolism of brain tissue and reduce the incidence of stroke when compared to patients in the low-risk group.

## Limitation

The present study has several limitations. Firstly, the sample size of this study was small, and further studies with large sample sizes are warranted. Secondly, this is a single-center study conducted on an Asian population with a high prevalence of ICAD. Therefore, there may be a selection bias in this sample, and the findings may be less applicable to other populations. Finally, long-term followed-up of patients is necessary to determine the impact on the prevention and recurrence of TIA patients. Further research on race and ethnicity is also necessary.

## Conclusion

Multimodal MRI may provide valuable insight into TIA patients. This study suggested that rMTT is higher in high-risk TIA patients and emphasizes the importance of considering hypertension, hyperhomocysteinemia, higher luminal stenosis degree, and greater NWI as high-priority factors. These findings contribute to the early diagnosis and individualized treatment of TIA patients, providing an additional clinical perspective.

## Data availability statement

The original contributions presented in the study are included in the article/supplementary material, further inquiries can be directed to the corresponding author.

## Ethics statement

The studies involving humans were approved by the First Hospital of Harbin Medical University Ethics Committee. The studies were conducted in accordance with the local legislation and institutional requirements. The participants provided their written informed consent to participate in this study. Written informed consent was obtained from the individual(s) for the publication of any potentially identifiable images or data included in this article.

## Author contributions

YS, JS, and WW conceived and designed the research. YS acquired the data. YS, JS, and YC analyzed and interpreted the data. YS and YC performed the statistical analysis. WW handled the supervision. YS drafted the manuscript. All authors contributed to the article and approved the submitted version.

## Conflict of interest

The authors declare that the research was conducted in the absence of any commercial or financial relationships that could be construed as a potential conflict of interest.

The reviewer BS declared a shared parent affiliation with the authors to the handling editor at the time of review.

## Publisher’s note

All claims expressed in this article are solely those of the authors and do not necessarily represent those of their affiliated organizations, or those of the publisher, the editors and the reviewers. Any product that may be evaluated in this article, or claim that may be made by its manufacturer, is not guaranteed or endorsed by the publisher.
